# Notes and News

**Published:** 1891-10-03

**Authors:** 


### NOTES AND NEWS.

Collected and Communicated.

Australia is just waking up to the advantages of cottage hospitals, and the rousing is chiefly due to the reports of two medical men who have lately visited England and America. Sydney is in advance of Melbourne in a practical experiment on small local hospitals. A "cottage hospital" established in the suburban municipality of Balmain has been most successful in every respect, and there is a tendency in the colony to multiply institutions of that sort. Small hospitals would be infinitely preferable to the huge crowded buildings of our colonial towns.

Miss Marjory Shanks Schaw, Glasgow, has given £40,000 as the nucleus for a convalescent home in connection with Glasgow Royal Infirmary. So there is some one who still has faith in this institution in spite of the disrepute its nurses have brought upon it. It will be a grand thing for the Royal to have its own convalescent home; we only wish the London Hospital had a chance of a home of its own somewhere at the sea. The Birmingham Hospital Saturday folk have been having a look at Llandudno with the thought of establishing a convalescent home there.

When Deputy-Surgeon-General James Cleghorn left Lucknow in August, to undertake the post of Inspector-General of the Civil Hospitals of the Punjaub, he was feasted and presented with a eulogistic address, signed by princes and all sorts of native grandees. Part of the address is worth quoting, if only to show the progress of hospital work at Lucknow, and how the movement for supplying medical aid to the native women is appreciated. "The Dufferin Hospital (Lady Lyall Branch), where *purdanashin* ladies by hundreds are being treated every day, and the Lying-in Hospital, where lying-in cases are specially treated, will be permanent monuments of your energy and devotion (as well as that of your sympathetic and devoted wife—may she live long!) to the welfare of our females. This is not all. You have in many other ways manifested your abiding interest for the physical welfare of our womankind. You have made special provision for the treatment of females in every hospital in the city, and opened a special Female Department in the King's Hospital. In short, you have throughout excellently advanced the noble movement inaugurated by her Excellency the Marchioness of Dufferin and Ava. One can have some idea of the vigilance shown by you during your administration to improve the efficiency of medical treatment from the table annexed below, showing the number of cases treated in the first year of your administration (1886) and the number in 1890:—

| | |
|---|---|
| In 1886 the number of outdoor patients was ... | 46,555 |
| Ditto indoor do. ... | 1,434 |
| Ditto major operations ... | 676 |
| Of this, females alone numbered ... ... ... | 10,382 |
| In 1890 the number of outdoor patients was ... | 183,314 |
| Ditto indoor do. ... | 2,390 |
| Ditto major operations ... | 945 |
| Of this, females alone numbered ... ... ... | 46,214" |

The report of the St. Helens Cottage Hospital just out expresses the pressing need for the enlargement of the institution, it having been lately often overcrowded. The number of patients admitted during the year was 231. There is a balance of £553 in hand, but this will not go far towards the necessary enlargement, and donations are badly needed.

The annual meeting of the Newcastle Hospital Sunday Fund Committee was held on September 17th. Ald. Stephens read the Secretary's annual report, which showed that there had been an increase in the receipts this year, the total being £4,647 12s. 7d., the largest amount that had ever been received by the fund in one year. The increase in church and chapel collections had been £31 1s., and the increase in the factory and workshop collections was £256 11s. 8d. The report was adopted and the usual votes of thanks passed.

The trailing of the coat is practised even in hospital matters in Ireland, where every opportunity for a row is regarded with more pleasure than aversion. A doctor is needed for Queenstown Dispensary, and lo! there is an election and two rival candidates, one a Catholic and one a Protestant, and the fight (of words) begins. The Matron of Clare Infirmary engages a Protestant as a probationer, and the thunder rumbles; the Matron herself is a Catholic, but she is also Irish and equal to a fight, so she stands by her choice, and threatens to resign. It is a pity that it is the question of religion which is usually chosen as a matter of difference, and we applaud the Matron of Clare.

Sir Whittaker Ellis opened Coleraine Cottage Hospital in August. It is a neat little building, after designs by Mr. W. J. Given, and should prove of service to the town. On the ground floor, to the left of the hall, is the committee-room, and behind this is the kitchen, pantry, &c. On the right there are several wards, handsomely furnished, which it is intended shall be used for men. Here also is a neat little surgery, provided with a number of useful appliances, and off the landing on the top of the wide staircase is an operating room, appropriately furnished, and other private rooms. There is a splendid bath, and several wards, all completely furnished, to be used for women. A Turkish bath has also been provided. The rooms are all well lighted.

Aberdeen is a lucky town; there dwell three ladies, Miss Rachel F. Lumsden, who is Hon. Superintendent of the nursing at the Royal Infirmary; Miss Katherine M. Lumsden, who is Hon. Superintendent of nursing at the Hospital for Sick Children; and Miss Lumsden of Camphill, a cousin of the first two, who till this year maintained a cottage home for the convalescent children. The home was always full, and this year Mr. Charles M'Donald presented the Committee with a larger cottage to hold eleven small convalescents, which is known as the Eidda Home. It is built almost on the moor, and well sheltered to the north and east. Nurse Annie, who is in charge, seems loved by her flock, and the luck of Aberdeen seems to have reached its consummation.

Mr. R. L. Stevenson has been writing an account of the lepers at Hawaii—a truthful account in which sentiment exists but does not predominate over sense. Here, for instance, is part of an account of the departure of a small band of lepers; "Between nine and ten of the same morning the schooner lay to off Hookena and a whale-boat came ashore. The village clustered on the rocks for the farewell—a grief perhaps, a performance certainly. We miss in our modern life these operatic consolations of the past. The lepers came singly and unattended; the elder first, the girl a little after, tricked out in a red dress and with a fine red feather in her hat. In this bravery, it was the more affecting to see her move apart on the rocks and crouch in her accustomed attitude. But this time I had seen her face; it was scarce horribly affected, but had a haunting look of an unfinished wood doll, at once expressionless and disproportioned; doubtless a sore spectacle in the mirror of youth."

